# OLDER ADULTS’ USE OF HOME AND COMMUNITY-BASED SERVICES: INDIVIDUAL ATTRIBUTES AND LOCATION DISPARITIES

**DOI:** 10.1093/geroni/igad104.1495

**Published:** 2023-12-21

**Authors:** Karen Roberto, Tina Savla

## Abstract

Home and Community-Based Services (HCBS) are designed to support older adults with functional and cognitive limitations. Yet, use of services is low, gaps exist in service provision, and available services do not always meet the person’s need. This symposium focuses on idiosyncrasies surrounding HCBS use. Nah and colleagues identify individual (income, ADL limitations, informal support) and county-level (poverty and public spending on services) factors associated with HCBS use among persons living with dementia in rural Appalachia. Shippee and colleagues’ examination of services received/desired indicates that older adults who are Black/other race/ethnicity were more likely to receive ADS; individuals living with AD/ADRD consumed personal care services at the highest proportion. Cheng’s and Li’s assessment of generosity in supporting HCBS reveals that while overall state support for HCBS has increased, Medicaid and OAA programs expenditures for services varies substantially across states. Fortinsky and colleagues’ examination of risk factors for emergency department use among older adult Medicaid HCBS users shows use was associated with high school education, more severe depressive symptoms, and more chronic conditions; non-Hispanic Asian individuals had the lowest likelihood of use. Konetzka and Wang provide national evidence that use of Medicaid HCBS influences Medicare post-acute care utilization among the dually enrolled. Collectively, the symposium highlights the importance of taking into consideration individual variations in care needs, local and state support for HCBS, and disparities in healthcare and service use influencing older adults’ use of HCBS, and advances understanding of intersecting micro- and macro-level effects on service availability, use, and outcomes.

